# The Biological Properties of the *FAS* and *TACR3* Genes and the Association of Single-Nucleotide Polymorphisms with Milk Quality Traits in Gannan Yak

**DOI:** 10.3390/foods14091575

**Published:** 2025-04-30

**Authors:** Tong Wang, Xiaoming Ma, Chaofan Ma, Qinran Yu, Chunnian Liang, Ping Yan

**Affiliations:** 1 Key Laboratory of Animal Genetics and Breeding on Tibetan Plateau, Ministry of Agriculture and Rural Affairs, Key Laboratory of Yak Breeding Engineering of Gansu Province, Lanzhou Institute of Husbandry and Pharmaceutical Sciences, Chinese Academy of Agricultural Sciences, Lanzhou 730050, China; 2 Institute of Western Agriculture, The Chinese Academy of Agricultural Sciences, Changji 931100, China; 3 Agricultural Genomics Institute at Shenzhen, Chinese Academy of Agricultural Sciences, Shenzhen 518000, China

**Keywords:** Gannan yak, *FAS* gene, *TACR3* gene, SNPs, milk quality traits

## Abstract

Fatty acid synthase (*FAS*) is a fundamental metabolic enzyme that catalyzes the synthesis of endogenous fatty acids; TACR3, also known as tachykinin receptor 3 or NK3R, is an important G-protein-coupled receptor that is primarily responsible for responding to neuropeptides such as neurokinin B (NKB) and plays a crucial role in embryonic development, organ formation, and cell differentiation. This study aimed to explore the association between the single-nucleotide polymorphisms (SNPs) of the *FAS* and *TACR3* genes and the milk quality of Gannan yak and to determine them as potential molecular marker loci for the milk quality of yaks. The genotyping of 162 Gannan yaks was performed using liquid-phase chip technology. Association analyses were conducted between the obtained SNP loci genotypes and milk composition traits, including milk protein, casein, non-fat solids, and acidity. Comparative sequence analysis of two genes (*FAS* and *TACR3*) across multiple species revealed that the yak *FAS* gene exhibited the highest homology with *Bos taurus* and *Bos indicus*, while the yak *TACR3* gene showed the greatest sequence similarity to *Bos taurus*. Hardy–Weinberg equilibrium tests were performed on four SNP loci, and the equilibrium indices of the four loci were 0.799, 0.368, 0.689, and 0.948 (*p* > 0.05), indicating that all of these loci are in Hardy–Weinberg equilibrium state. g.13,276T>C (*FAS*) was significantly correlated with lactose content traits (*p* < 0.05); g.74,382C>G (*FAS*) was significantly correlated with casein, protein, total solids, non-fat solids, and acidity traits (*p* < 0.05); g.40,529A>G (*TACR3*) was significantly correlated with protein, non-fat solids, citric acid, and acidity traits (*p* < 0.05). The influence of g.40,555C>T (*TACR3*) on these traits did not reach a significant level (*p* > 0.05). This study suggests that two genes can serve as potential candidate genes affecting the quality of Gannan yak milk, providing reference genes for improving the quality of Gannan yak milk.

## 1. Introduction

The yak is known as the ‘boat of the plateau’ and the ‘omnipotent livestock’ because of its strong adaptability to harsh environments such as low temperatures and low oxygen. Yak milk, as one of the important sources of nutrition for herdsmen, provides important substances such as high-quality protein, minerals, amino acids, and fatty acids [1]. Long-term living in a special environment gives yak milk a certain uniqueness. Compared with other milks, yak milk has more abundant nutrients [2]. Among them, the fat content is higher than that of the milk of Holstein cows [3], and the lactose content is also higher than that of ordinary milk, which is close to human milk [4]. In addition, the calcium and phosphorus ratio of yak milk is also close to that of human milk, which is more conducive to human absorption [5]. Yak milk contains low β-lactoglobulin, so yak milk is also regarded as ‘the best in milk’, with low sensitization [6]. In addition to the nutritional value, yak milk is rich in n-3 polyunsaturated fatty acids, which can also improve the body’s immune ability [7]. Yak milk also contains a variety of immune active substances [8], which have antioxidant and antibacterial properties and regulate the intestinal flora [9,10,11]. In recent years, with the development of the economy and the improvement in people’s living standards, people’s attention to diet has increased, the attention to dairy products has also increased, and the demand for high-quality milk sources is increasing day by day. Yak milk has high nutritional value and is a high-quality raw material for the production of dairy products. It is also the main resource for the development of the characteristic dairy industry in the plateau area. Yak milk has high development and application value, which can meet the nutritional needs of consumers of different ages. Milk quality traits include protein, fat, and lactose, which are quantitative traits and are affected by multiple minor genes [12]. Studying the functional genes of milk quality traits can directly affect the quality of dairy products and the development of the industry.

Genetic markers are often used to identify genetic differences, among which molecular genetic markers are the most widely used [13]. Genotyping based on DNA molecular markers has become an important technical means in the field of modern animal breeding. Single-nucleotide polymorphisms (SNPs) have become the most widely used analytical marker due to their unique biological characteristics [14]. They are ubiquitous in human and animal genomes, with an average of one SNP per 500–1000 base pairs [15]. SNPs are distributed throughout the genome. SNPs in the coding region can be divided into synonymous protein-encoding SNPs and missense protein-encoding SNPs. Missense protein-encoding SNPs can change the amino acid sequence and affect the related functions of proteins [16,17]. Many studies have shown that gene polymorphisms can affect milk production traits. Viale [18] found 45 SNP loci in 32 genes of Holstein–Friesian bulls, of which 14 SNP loci have significant effects on the milk production performance of dairy cows. In the study of Liu [19], 12 single-nucleotide polymorphisms of the *KLF6* gene were identified to be significantly correlated with milk yield, fat percentage, and protein in the first and second lactation periods. Selionova [20] studied the milk quality traits of Karachai goats by genome-wide association analysis, identified 167 significant SNPs related to the milk composition of Karachai goats, and found genes related to dry matter weight and fatty acids. Zwierzchowski [21] found new single-nucleotide polymorphisms in the 5′ regulatory region of the *SLC5A1* gene and found that these loci were associated with milk yield and milk composition in Holstein cows. At present, there is still a certain gap between China’s dairy industry and that of other countries with more developed animal husbandry, and there is still room for progress.

Fatty acid synthase (FAS) is a basic metabolic enzyme that catalyzes the synthesis of endogenous fatty acids. It catalyzes the synthesis of palmitic acid (16:0) by acetyl-CoA and malonyl-CoA [22]. It has been found that the human fatty acid synthase gene is located on 17q25 (encoding 7536bp cDNA sequence), the bovine gene is located on 19q22, and the chicken gene is located on chromosome 18. Under normal physiological conditions, the expression of *FAS* is tightly regulated by many factors such as the environment, hormones, and nutrition [23]. For example, in the uterus and breast tissues, the expression of *FAS* is regulated by estrogen and progesterone, and sex hormones can promote the synthesis of *FAS* [24,25]. However, how estrogen regulates *FAS* in normal uterine and breast tissues is not entirely clear. Studies have shown that *FAS* in mammals not only affects fatty acid metabolism in adult animals but also plays a role in embryonic development [26] and synthesizes milk fat during lactation [27]. Chris A. Morris [28] found that FAS affects adipose tissue and milk fat percentage in animals. Tachykinin receptor 3 (*TACR3*) belongs to the G-protein-coupled receptor family, which mainly binds to neurokinin B (NKB) and participates in neuroendocrine regulation [29]. It is located on sheep chromosome 8 and encodes 465 amino acids [30]; it is located on chromosome 8 of pigs. The loss of *TACR3* or NKB function in the human hypothalamus can lead to congenital gonadal dysplasia and infertility, indicating that human *TACR3* plays an important role in reproductive function [31]. Yang [32] found that after knocking out the *TACR3* gene in female mice, the uterus weight of the mice was lower, the estrus was abnormal, and the litter size per litter was less in adulthood. *TACR3* was also found to be expressed in the ovaries and is associated with ovarian function and follicular development [33]. The TACR3/TAC3 system plays a key role in regulating gonadotropin secretion and sex hormone feedback regulation of the reproductive axis [34]. Therefore, in this study, 162 Gannan yaks were selected as samples, the *FAS* and *TACR3* genes were used as candidate genes for milk quality traits, and their associations with milk quality traits were analyzed to provide a theoretical basis for the breeding of Gannan yaks.

## 2. Materials and Methods

### 2.1. Ethics Statement

All animal experiments mentioned in this study were approved by the Animal Ethics Committee of Lanzhou Institute of Animal Husbandry and Veterinary Medicine, Chinese Academy of Agricultural Sciences, approval number: 1610322020018.

### 2.2. Experimental Animal Selection

In this study, 162 lactating Gannan yaks selected from Xiahe County, Gannan Tibetan Autonomous Prefecture, Gansu Province, were used as experimental subjects. Geographical coordinates: 34°32′–35°34′ N, 101°54′–103°25′ E; altitude: 3000–3800 m. The selection criteria included the following: calving parity of 2–3 times and no history of mastitis or reproductive system diseases, confirmed by the detection of three estrous cycles in the local animal husbandry station. The average daily milk yield is the group mean. In this study, we tried to control for controllable environmental factors as much as possible, such as selecting 162 Gannan yaks with similar physical conditions, all from the same grazing area, and without additional feed supplementation. We ensured relatively consistent feeding environment, breeding conditions, and climate conditions. In order to reduce the interference of biological rhythms on the experimental results, all test samples were collected in a single daytime period.

### 2.3. Sample Collection and DNA Extraction

In this study, 162 healthy female Gannan yaks were selected as experimental subjects, and their ear tissues and milk samples were systematically collected for genomics and milk composition analysis. The ear tissue samples were obtained with sterile pliers at the leading edge of the right ear. Immediately after collection, they were immersed in liquid nitrogen, transported to the laboratory, sub-packed into a freezer tube, and stored in an ultra-low-temperature refrigerator at −80 °C until use for analysis. Genomic DNA was extracted by the MGIEasy magnetic bead kit (DP341, Tiangen Biochemical Technology Co., Ltd., Beijing, China). The obtained DNA was detected by the Qubit fluorescence quantitative instrument, with 1% agarose gel electrophoresis to evaluate the integrity and Nanodrop to determine the purity. All qualified samples were used to construct the DNA library. Milk sample collection was strictly controlled at the same time period. Before the operation, 0.1% iodophor was used to disinfect yak breasts. After breast massage stimulation, the first three milk samples were discarded to eliminate the risk of pollution. The intermediate milk segment was collected in 50 mL sterile centrifuge tubes, and the individual ear number, altitude, and sampling time were marked. The samples were transferred to the laboratory at a constant temperature of 4 °C for milk quality analysis.

### 2.4. Genotyping

In this study, high-throughput genotyping technology (cGPS) based on target sequence liquid hybridization capture was used to detect genome-wide single-nucleotide polymorphism markers in 162 Gannan yaks. The DNA sample was digested by the fragment enzyme, and the A base was added to the 3′ end; T4 ligase was used to connect the sequencing adapter and the DNA fragment, and the product was purified. The purified product was subjected to PCR amplification; the probe was added to the hybridization reagent for the hybridization reaction, the target region and the product were captured, the non-specific binding fragment was removed, and PCR amplification was performed again to complete the sequencing library construction. The Qubit fluorescence quantitative instrument was used for library determination and quality inspection. After qualification, sequencing chip loading and sequencing were performed. Fastp was used to perform quality control on the raw reads data (filter low-quality reads; remove the connector fragment in the reads; filter excessive N-base reads; reads with filter length less than 100). Using the alignment software BWA, the filtered clean reads were compared with the reference genome Bosgru v3.0 (GCA_005887515.1) to locate the location of clean reads on the reference genome. Based on the clean reads reference genome sequence alignment results, the genotype of the target site was detected by the mutation analysis software GATK mutation detection tool HaplotypeCaller. Genotypes were judged according to the proportion of supported reads of different allele sites. When the proportion of mutant reads supported was ≥0.8 or ≤0.2, the site was judged to be a homozygous genotype. When the proportion of mutant reads supported was between 0.2 and 0.8, it was judged to be a heterozygous genotype. Finally, ANNOVAR software (v2025-03-21) was used to annotate the detected gene variations.

### 2.5. Analysis of Milk Composition of Gannan Yak

Referring to NY/T 2659-2014, routine nutritional indexes, such as casein, protein, fat, total solids, non-fat solids, lactose, average diameter of fat globule, and acidity, of the milk of 162 Gannan yaks were determined by a multifunctional milk composition rapid analyzer (MilkoScanTM FT120)(Danish FUCHS Analytical Instruments Ltd., Hellerup, Denmark).

### 2.6. Association Analysis Between Different SNP Loci and Dairy Quality Traits

We matched the genotype of each Gannan yak with its dairy quality traits one by one. We imported the data into SPSS 25(IBM, Armonk, NY, USA) for normality testing and used the genotype of each SNP as an independent grouping variable. Genotype was used as a factor, and milk quality traits were used as the dependent variable. We used one-way ANOVA to analyze the association between different SNP loci and dairy quality traits.

### 2.7. Bioinformatics Analysis

MegAlign (7.1.0) software was used to compare the homology of the FAS and TACR3 gene CDS sequences of seven species downloaded from NCBI, including *Bos mutus*, *Bos taurus*, *Bos indicus*, *Homo sapiens*, *Bubalus bubalis*, *Ovis aries*, and *Sus scrofa*. MEGA11 software was used to construct the phylogenetic trees of different species. The physicochemical properties and protein structure of the two proteins were analyzed using public online such as NCBI (https://www.ncbi.nlm.nih.gov/orffinder/, accessed on 1 March 2025), ProtParam (https://web.expasy.org/protparam/, accessed on 1 March 2025), ProtScale (https://web.expasy.org/protscale/, accessed on 1 March 2025), SignalP (https://services.healthtech.dtu.dk/services/SignalP-6.0/, accessed on 1 March 2025), TMHMM (https://services.healthtech.dtu.dk/services/TMHMM-2.0/, accessed on 1 March 2025), Psort (https://wolfpsort.hgc.jp/, accessed on 1 March 2025), NetPhos (https://services.healthtech.dtu.dk/services/NetPhos-3.1/, accessed on 1 March 2025), and STRING (https://cn.string-db.org/, accessed on 1 March 2025).

### 2.8. Data Statistical Analysis

GDICALL (http://www.msrcall.com/gdicall.aspx, accessed on 1 March 2025) online software was used to calculate the expected He (expected heterozygosity), observed Ho (observed heterozygosity), PIC (polymorphism information content), genotypic frequencies, allelic frequencies, and p value of the Hardy–Weinberg test at different sites of the *FAS* and *TACR3* genes. Subsequently, one-way analysis of variance (ANOVA) was performed on the association between *FAS* and *TACR3* gene polymorphisms and milk quality traits using IBM SPSS Statistics 25 (IBM, Armonk, NY, USA), and the results are presented in the form of the mean ± standard deviation. The statistical significance level was set as follows: *p* < 0.05 indicates a significant difference, and *p* < 0.01 indicates an extremely significant difference.

## 3. Results

### 3.1. Homology Analysis and Phylogenetic Tree Construction of FAS and TACR3 Genes

**Figure 1:**
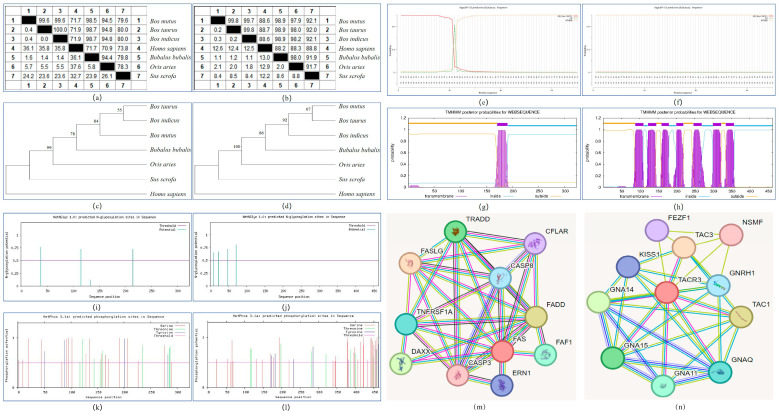
Bioinformatics analysis of FAS and TACR3 genes and proteins in Gannan yak. (**a**,**b**) Gene sequence homology alignment; (**c**,**d**) construction of gene phylogenetic tree; (**e**,**f**) protein signal peptide prediction; (**g**,**h**) protein transmembrane domain prediction analysis; (**i**,**j**) protein glycosylation site prediction; (**k**,**l**) prediction of potential phosphorylation sites of protein; (**m**,**n**) protein interaction network prediction.

The amino acid sequences of the *FAS* and *TACR3* genes in different species were downloaded from the NCBI database and analyzed. The results are shown in Figure 1. The CDS sequences of the *FAS* and *TACR3* genes in *Bos mutus*, *Bos taurus*, *Bos indicus*, *Homo sapiens*, *Bubalus bubalis*, *Ovis aries*, and *Sus scrofa* were compared by MegAlign software. For the *FAS* gene, the highest homology with *Bos mutus* was *Bos taurus* and *Bos indicus* (99.6%), the lowest was *Homo sapiens* (71.7%), and the homology with *Bubalus bubalis*, *Ovis aries*, and *Sus scrofa* was 98.5%, 94.5%, and 79.6%, respectively. Among the *TACR3* genes, *Bos taurus* had the highest homology with *Bos mutus* (99.8%) and the lowest homology with *Homo sapiens* (88.6%). The homology with *Bos indicus*, *Bubalus bubalis*, *Ovis aries*, and *Sus scrofa* was 99.7%, 98.9%, 97.9%, and 92.1%, respectively. In the phylogenetic tree of the *FAS* gene, *Bos indicus* was first clustered with *Bos taurus*, then with Bos mutus, *Bubalus bubalis*, *Ovis aries*, *Sus scrofa*, and finally with *Homo sapiens*. In the phylogenetic tree of the *TACR3* gene, *Bos mutus* was first clustered with *Bos taurus*, then with *Bos indicus*, *Bubalus bubalis*, *Ovis aries*, *Sus scrofa*, and finally with *Homo sapiens*.

### 3.2. Physicochemical Properties, Hydrophilicity/Hydrophobicity, Signal Peptide, Transmembrane Domain, and Advanced Structure Analysis of FAS and TACR3 Proteins

The physical and chemical properties of yak FAS and TACR3 proteins were analyzed by ProtParam online software, finding the following properties: two protein molecular formulas (C_1548_H_2492_N_460_O_497_S_29_ and C_2397_H_3672_N_610_O_643_S_23_), the total number of atoms (5026 and 7345), the relative molecular mass (36,429.34 and 52,060.61), the theoretical isoelectric point (6.63 and 9.41), the instability coefficient (32.93 and 43.73), the protein fat solubility index (73.07 and 93.13), the total average hydrophilicity index (−0.637 and 0.220), and the estimated half-life (30 h and 30 h). This shows that FAS and TACR3 are hydrophilic proteins and hydrophobic proteins, respectively. This study used SignalP5.0 online software to predict the protein signal peptide. The results are shown in Figure 1e,f. The FAS protein has a signal peptide between the 22nd and 23rd amino acids, and there is no signal peptide in the TACR3 protein. In this study, TMHMM 2.0 online software was used to predict and analyze the transmembrane domains. It was found that there were one and seven transmembrane domains in the FAS and TACR3 proteins, respectively. The results are shown in Figure 1g,h. The subcellular localization of the FAS protein was predicted by Psort software. The results showed that the scores of the FAS protein in the plasma membrane, extracellular, nucleus, and mitochondria were 19, 11, 1, and 1, respectively. The TACR3 protein is mainly located on the plasma membrane, and its score was 32. The amino acid sequences of the two proteins were input into NetNGlyc online software to predict their glycosylation sites. In the FAS protein, the glycosylation sites NITE, NCTR, and NLTD were found at positions 38, 115, and 215, respectively. The glycosylation potential values of each site were 0.7636, 0.7271, and 0.7213, respectively. In the TACR3 protein, the glycosylation sites NWTN, NLSA, NLSA, and NITN were found at positions 9, 22, 48, and 71, respectively. The glycosylation potential values of each site were 0.6546, 0.6692, 0.7184, and 0.8014, respectively. Using NetPhos 3.1 to predict the potential phosphorylation sites of the protein, it was found that there were 30 and 46 potential phosphorylation sites in the two proteins, respectively. The results are shown in Figure 1k,l. Using the STRING online software to construct the FAS and TACR3 protein interaction network, it was found that the number of interaction network nodes was 11, and there may be interactions with many proteins. The results are shown in Figure 1m,n.

### 3.3. Genotype and Allele Frequencies and Polymorphism Information Content of FAS and TACR3 Genes at Different Loci

**Figure 2:**
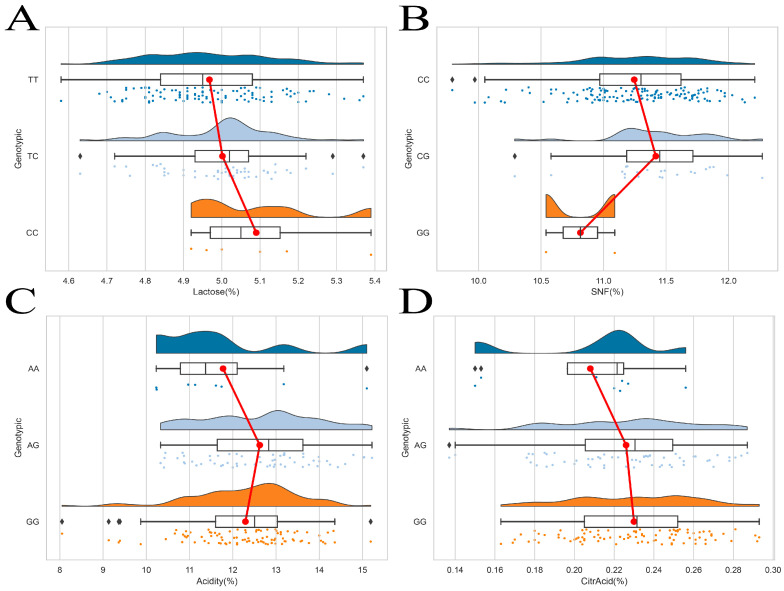
Genotypes of *FAS* and *TACR3* loci and distribution of milk quality traits. (**A**) g.13,276T>C; (**B**) g.74,382C>G; (**C**,**D**) g.40,529A>G. The small dots of different colors in the figure represent the values and distribution of milk quality traits corresponding to different genotypes of Gannan yak. The red line represents the connection of the average values of milk quality traits corresponding to three different genotypes.

Figure 2 shows the distribution of genotypes and milk quality traits at different loci of the *FAS* and *TACR3* genes. The results showed that the distribution of milk quality traits corresponding to different genotype loci of the two genes was more uniform. The genotype frequency, allele frequency, theoretical heterozygosity, observed heterozygosity, and polymorphic information content of different loci of the two genes were further calculated. The results are shown in Table 1. The four loci of the two genes were all located in the intron region. The genotype frequencies of TT and CC, which were 0.636 and 0.846, respectively, were the highest in the two loci of the *FAS* gene, indicating that the two loci of the *FAS* gene were mainly homozygous. Among the two loci of the *TACR3* gene, the genotype frequencies of GG and CC, both of which were 0.580, were the highest. The gene frequencies of T and C at the two sites of the *FAS* gene were 0.799 and 0.917, respectively, indicating that the unmutated alleles at these two sites were dominant. Among the two loci of the *TACR3* gene, the highest gene frequencies were 0.765 (G) and 0.762 (C). Among the four loci of the *FAS* (g.13,276T>C and g.74,382C>G) and *TACR3* (g.40,529A>G and g.40,555C>T) genes, the homozygosity (0.679, 0.847, 0.641, and 0.638) was greater than the heterozygosity (0.321, 0.153, 0.359, and 0.362). The polymorphic information content (PIC) of g.13,276T>C, g.74,382C>G, g.40,529A>G and g.40,555C>T was 0.269, 0.141, 0.295, and 0.297, respectively. Usually, a PIC value greater than 0.25 is considered to be moderate polymorphism, and a value greater than 0.5 is considered to be high polymorphism. According to this standard, loci g.13,276T>C, g.40,529A>G, and g.40,555C>T were classified as moderately polymorphic, and locus g.74,382C>G was less polymorphic, indicating that the genetic variation in these loci in the population is at a medium level. The Hardy–Weinberg equilibrium test was performed on the four SNP sites, and the equilibrium indexes of the four sites were 0.799, 0.368, 0.689, and 0.948 (*p* > 0.05), indicating that these sites were in Hardy–Weinberg equilibrium.

### 3.4. Association Analysis of FAS and TACR3 Gene Polymorphisms with Milk Quality Traits in Gannan Yaks

The analysis of the impact of *FAS* and *TACR3* gene polymorphisms on dairy quality traits in Gannan yaks shows that different genotypes of these loci have significant genotype effects on dairy quality traits, as shown in Table 2. Firstly, in the g.13,276T>C locus of the *FAS* gene, the effects of different genotypes on casein, protein, fat, total solids, non-fat solids, lactose, citric acid, and acidity did not reach significant levels (*p* > 0.05). However, in terms of the content of casein, protein, fat, and total solids, the mutant heterozygous genotype TC is higher than the wild-type homozygous genotype TT and the mutant homozygous genotype CC. This indicates that at this locus, genotype mutations have a positive effect on the milk quality of Gannan yaks. The g.74,382C>G locus of the *FAS* gene is significantly correlated with the casein, protein, non-fat solids, and acidity traits (*p* < 0.05), and the casein, protein, non-fat solids, and acidity contents of the mutant heterozygous genotype CG are significantly higher than those of the mutant homozygous genotype GG (*p* < 0.05). However, the effect of the g.74,382C>G site on fat, total solids, lactose, and citric acid did not reach a significant level (*p* > 0.05). The g.40,529A>G locus of the *TACR3* gene is significantly correlated with protein and non-fat solids (*p* < 0.05), and the content of the mutant heterozygous genotype AG is significantly higher than that of the wild homozygous genotype AA (*p* < 0.05). However, compared to several other dairy quality traits, it did not reach a significant level (*p* > 0.05). The different genotypes of the *TACR3* gene g.40,555C>T locus did not show significant effects on casein, protein, fat, total solids, non-fat solids, lactose, citric acid, and acidity (*p* > 0.05). The heterozygous mutant genotype CT has higher levels of casein, protein, non-fat solids, and acidity than the other two genotypes; the fat, total solids, and lactose contents of the homozygous mutant genotype TT are higher than those of the other two genotypes.

### 3.5. Linkage Disequilibrium Analysis of SNPs in FAS and TACR3 Genes

**Figure 3:**
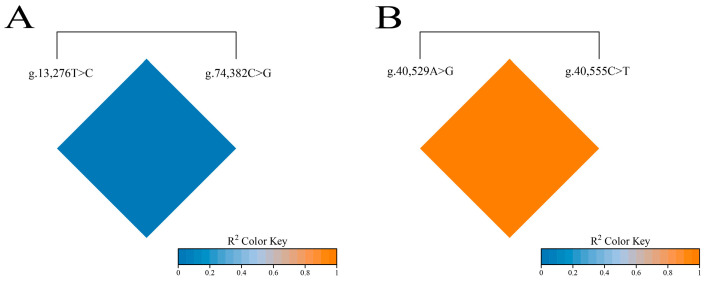
Linkage disequilibrium analysis between SNPs of *FAS* and *TACR3* genes. (**A**) Linkage disequilibrium analysis of two different loci in *FAS* gene. (**B**) Linkage disequilibrium analysis of two different loci in *TACR3* gene.

This study utilized an online tool (https://www.bioinformatics.com.cn/, accessed on 1 March 2025) to perform linkage disequilibrium analysis on four loci of the Gannan yak *FAS* gene (g.13,276T>C and g.74,382C>G) and *TACR3* gene (g.40,529A>G and g.40,555C>T), with the detailed results presented in Figure 3. The analysis indicated that there is complete linkage equilibrium between the g.13,276T>C and g.74,382C>G loci of the *FAS* gene, while complete linkage disequilibrium exists between the g.40,529A>G and g.40,555C>T loci of the *TACR3* gene.

## 4. Discussion

With the development of the social economy, people are paying more and more attention to their own health problems, and the demand for food is changing from food and clothing to nutrition and health. As a characteristic livestock species in the Qinghai–Tibet Plateau and its surrounding areas, the industrial chain formed by yak milk and other products has developed into the main industry and pillar industry of animal husbandry in alpine pastoral areas [36]. In the Qinghai–Tibet Plateau, yak milk accounts for more than 90% of milk consumption, indicating that yak milk is not only one of the sources of nutrition for herdsmen but also a source of income [37]. Because of the particularity of this geographical location, yak milk has formed high-quality characteristics and a unique flavor. In addition to the nutrients mentioned in the preface, yak milk is also rich in conjugated linoleic acid, which is called ‘natural concentrated milk’ [38]. Compared with ordinary milk, yak milk is characterized by a high concentration, high specific gravity, and high content. It has been identified that yak milk is one of the milk sources with the closest biological characteristics to human breast milk [39]. In the past, nearly 70% of yak milk was used to feed calves and herders themselves. In recent years, the market for yak milk has gradually developed. Compared with other milk, yak milk is a high-value commodity with great development potential [40]. The nutritional composition and quality safety of yak milk have begun to receive widespread attention from society. At present, there are mainly yak milk products such as yak yogurt, yak milk powder, casein, and bioactive peptides, but they are still in the initial stage of product development, with great development potential and broad prospects.

*FAS* contains two subunits, each containing seven different functional domains, in which the TE (thioesterase) domain plays a key role in the function of the *FAS* gene. Through phylogenetic tree and sequence homology comparison, it was found that the homology of the *FAS* gene is relatively conservative, which is consistent with the results of Ding [41]. Milk proteins and fat are the most important nutritional components in milk. The results of this study revealed that the g.74,382C>G locus of the *FAS* gene and the g.40,529A>G locus of the *TACR3* gene have a significant impact on the protein content in Gannan yak milk. Furthermore, both genes demonstrated that the protein content in heterozygous genotype yaks is higher than that in the two homozygous genotype yaks. The g.74,382C>G locus of the *FAS* gene also has a significant effect on the casein content in Gannan yak milk, with heterozygous genotypes exhibiting higher levels compared to homozygous genotypes. Casein is a phosphorus-containing protein that includes all eight essential amino acids required by the human body and is the most abundant protein found in cow’s milk [42]. The proportions of various casein fractions in the total casein of yak milk differ from those in ordinary cow’s milk, particularly in terms of α_s1_-CN and β-CN, which also have distinct functions. Yak milk effectively supplements essential components such as dry matter, fat, and lactose in the human diet. Additionally, its immunoglobulins and various minerals can enhance the immune system [43]. The g.74,382C>G locus of the *FAS* gene and the g.40,529A>G locus of the *TACR3* gene had an effect on the content of non-fat solids in Gannan yak milk. As a key enzyme in fat synthesis, fatty acid synthase (*FAS*) has been found to be expressed in mammary gland tissues during lactation [44]. A study on GWAS of Holstein milk fatty acids in China showed that *FAS* mainly affects medium-chain saturated fatty acids such as C10:0, C12:0, and C14:0 in Holstein cattle [45]. In tissues other than the mammary gland in mammals, the primary product of fatty acid synthesis is palmitic acid; however, in mammary gland tissues, FAS can specifically produce short-chain and medium-chain fatty acids. Roy et al. [46] used the fluorescence in situ hybridization and somatic cell hybridization techniques to locate the bovine FAS gene on chromosome 19. They confirmed the presence of numerous quantitative trait loci (QTLs) that influence milk fat content on this chromosome. Furthermore, they found that the polymorphic sites of the *FAS* gene were significantly associated with milk fat content, suggesting that the *FAS* gene is a candidate gene influencing milk fat content in cattle. Chris et al. [28] identified a QTL on chromosome 19 in cattle through linkage analysis and identified *FAS* as a candidate gene for this QTL. Five SNPs were detected and genotyped in three different cattle populations. Correlation analysis showed that the SNPs of the *FAS* gene were associated with fat and milk fat. Schennink et al. [47] found two significant SNPs (g.16,024G>A and g.17,924A>G) in a study of 1905 Holstein dairy herds, with the latter having a significant impact on milk fat percentage (*p* < 0.05). Laura et al. [48] reported an SNP (g.763G>C) in the 5′ flank region of bovine *FAS* which was significantly correlated with milk fat content in cows. The SNP altered the in vitro activity of the bovine *FAS* promoter and the Sp1/Sp3 binding ability of the sequence, thereby affecting the milk fat content. Based on the above research, the *FAS* gene can serve as a potential candidate gene for dairy quality traits. The expression of the TACR3/TAC3 system can promote the secretion of GnRH, playing a facilitating role in the seasonal reproductive regulation of the Syrian hamster [49]. However, reports on the influence of *TACR3* on milk quality traits in animals are scarce. Therefore, further in-depth studies are needed to investigate the mechanisms by which the *TACR3* gene affects milk quality in animals. Non-fat solids in milk refer to the sum of all solid components except fat in milk. Their existence is of great significance to the nutritional value, processing characteristics, and quality stability of dairy products. They are an important index to measure the quality of milk and directly affect the nutritional rating of dairy products. And the protein in the milk forms an emulsified structure through hydration, giving the dairy product a smooth texture. The acidity value of milk is one of the key indicators to measure the quality and safety of dairy products. Yak milk, with its high acidity and unique composition, has shown significant advantages in antibacterial and metabolic regulation [9]. It can be seen that the g.74,382C>G and g.40,529A>G loci have an effect on the acidity value, and the heterozygous genotype is higher than the homozygous genotype. Although several gene loci mentioned in this study are located in the intron region, they also play an important role [50]. And studies have shown that this region also has a certain impact on gene expression [51]. The results suggest that the *FAS* and *TACR3* genes can be used as potential molecular markers affecting the quality of yak milk, which provides a theoretical basis for improving the quality-related traits of yak milk. At the same time, it is also of great practical significance to study how the nutritional composition and quality of yak milk can be improved through *FAS* and *TACR3* gene regulation.

## 5. Conclusions

This study is the first to discuss the association between the polymorphisms at different loci of the *FAS* and *TACR3* genes and milk quality-related traits in yaks. The results indicate that several loci significantly affect the levels of relevant indicators in Gannan yak milk. Therefore, we hypothesize that these two genes can be considered candidate genes for regulating milk quality traits in yaks. However, the specific regulatory mechanisms require further in-depth investigation, and the findings have positive implications for improving yak milk quality.

## Figures and Tables

**Table 1:** The genotype and allele frequencies and polymorphism information content of different mutation sites of the *FAS* and *TACR3* genes were analyzed.

Gene	SNPs	Position	Genotypic Frequencies			Allelic Frequencies		He	Ho	X^2^	PIC	HW *p* Value
*FAS*	g.13,276T>C	Intron	TT	TC	CC	T	C	0.321	0.679	0.065	0.269	0.799
			0.636	0.327	0.037	0.799	0.201					
	g.74,382C>G	Intron	CC	CG	GG	C	G	0.153	0.847	0.810	0.141	0.368
			0.846	0.142	0.012	0.917	0.083					
*TACR3*	g.40,529A>G	Intron	AA	AG	GG	A	G	0.359	0.641	0.160	0.295	0.689
			0.049	0.370	0.580	0.235	0.765					
	g.40,555C>T	Intron	CC	CT	TT	C	T	0.362	0.638	0.004	0.297	0.948
			0.580	0.364	0.056	0.762	0.238					

Note: He: heterozygosity; Ho: homozygosity; X^2^: the chi square value, a statistical measure used to test whether a population follows the Hardy–Weinberg equilibrium; PIC: polymorphism information content. PIC < 0.25 indicates low polymorphism, 0.25 < PIC < 0.5 indicates moderate polymorphism, and PIC > 0.5 indicates high polymorphism. A HW *p* value > 0.05 indicates that the population’s genes are in Hardy–Weinberg equilibrium, suggesting that the samples originated from the same Mendelian population [35].

**Table 2:** Association analysis of *FAS* and *TACR3* gene SNPs with milk quality traits in Gannan yaks.

**FAS g.13,276T>C**								
**Genotype**	**Casein/%**	**Protein/%**	**Fat/%**	**TS/%**	**SNF/%**	**Lactose/%**	**Citric Acid/%**	**Acidity/°T**
TT	4.11 ± 0.28	4.92 ± 0.36	5.24 ± 2.20	16.66 ± 2.54	11.25 ± 0.46	4.97 ± 0.16	0.23 ± 0.34	12.44 ± 1.19
TC	4.12 ± 0.24	4.95 ± 0.33	5.30 ± 1.76	16.48 ± 1.67	11.39 ± 0.36	5.00 ± 0.12	0.23 ± 0.30	12.53 ± 1.26
CC	3.93 ± 0.35	4.75 ± 0.48	3.41 ± 0.37	14.12 ± 1.22	11.20 ± 0.51	5.09 ± 0.17	0.22 ± 0.28	13.03 ± 0.93
*p* value	*p* = 0.274	*p* = 0.414	*p* = 0.202	*p* = 0.051	*p* = 0.144	*p* = 0.104	*p* = 0.424	*p* = 0.627
**FAS g.74,382C>G**								
**Genotype**	**Casein/%**	**Protein/%**	**Fat/%**	**TS/%**	**SNF/%**	**Lactose/%**	**Citric Acid/%**	**Acidity/°T**
CC	4.08 ± 0.26 ^a^	4.90 ± 0.36 ^a^	5.21 ± 2.10	16.35 ± 2.09	11.27 ± 0.45 ^ab^	5.00 ± 0.16	0.23 ± 0.33	12.34 ± 1.22 ^a^
CG	4.24 ± 0.27 ^a^	5.10 ± 0.33 ^a^	5.39 ± 1.94	16.73 ± 1.94	11.47 ± 0.37 ^a^	4.93 ± 0.15	0.24 ± 0.22	13.01 ± 1.22 ^a^
GG	3.58 ± 0.24 ^b^	4.35 ± 0.38 ^b^	3.34 ± 0.78	13.94 ± 0.40	10.82 ± 0.39 ^b^	5.03 ± 0.57	0.25 ± 0.24	10.82 ± 0.14 ^b^
*p* value	*p* = 0.001	*p* = 0.004	*p* = 0.412	*p* = 0.184	*p* = 0.043	*p* = 0.162	*p* = 0.170	*p* = 0.010
**TACR3 g.40,529A>G**								
**Genotype**	**Casein/%**	**Protein/%**	**Fat/%**	**TS/%**	**SNF/%**	**Lactose/%**	**Citric Acid/%**	**Acidity/°T**
AA	4.04 ± 0.41	4.72 ± 0.53 ^b^	6.96 ± 3.21	17.96 ± 3.28	10.99 ± 0.60 ^b^	5.02 ± 0.21	0.21 ± 0.37	11.78 ± 1.64
AG	4.18 ± 0.27	5.02 ± 0.37 ^a^	5.79 ± 2.78	17.05 ± 2.72	11.38 ± 0.43 ^a^	4.98 ± 0.16	0.23 ± 0.34	12.62 ± 1.31
GG	4.05 ± 0.26	4.84 ± 0.39 ^ab^	5.30 ± 2.49	16.39 ± 2.41	11.21 ± 0.48 ^ab^	4.99 ± 0.15	0.23 ± 0.31	12.29 ± 1.23
*p* value	*p* = 0.127	*p* = 0.008	*p* = 0.169	*p* = 0.113	*p* = 0.026	*p* = 0.714	*p* = 0.175	*p* = 0.147
**TACR3 g.40,555C>T**								
**Genotype**	**Casein/%**	**Protein/%**	**Fat/%**	**TS/%**	**SNF/%**	**Lactose/%**	**Citric Acid/%**	**Acidity/°T**
CC	4.03 ± 0.28	4.84 ± 0.39	5.19 ± 2.25	16.39 ± 2.41	11.21 ± 0.48	4.99 ± 0.15	0.23 ± 0.31	12.29 ± 1.23
CT	4.17 ± 0.26	5.01 ± 0.36	5.62 ± 2.50	17.04 ± 2.74	11.37 ± 0.42	4.98 ± 0.16	0.23 ± 0.35	12.58 ± 1.27
TT	4.11 ± 0.43	4.84 ± 0.61	6.79 ± 3.05	17.90 ± 3.07	11.13 ± 0.69	5.00 ± 0.21	0.21 ± 0.38	12.16 ± 1.92
*p* value	*p* = 0.136	*p* = 0.170	*p* = 0.124	*p* = 0.114	*p* = 0.105	*p* = 0.934	*p* = 0.281	*p* = 0.351

Note: in the same set of data, different lowercase letters indicate significant differences (*p* < 0.05), and the data are expressed as the mean ± standard deviation.

## Data Availability

The original contributions presented in this study are included in the article. Further inquiries can be directed to the corresponding authors.
